# The different colorectal tumor risk related to GLP-1 receptor agonists and SGLT2 inhibitors use: a network meta-analysis of 68 randomized controlled trials

**DOI:** 10.1097/JS9.0000000000003450

**Published:** 2025-09-11

**Authors:** Chao-Ming Hung, Bing-Yan Zeng, Chih-Wei Hsu, Po-Huang Chen, Cheuk-Kwan Sun, Andre F. Carvalho, Brendon Stubbs, Yen-Wen Chen, Tien-Yu Chen, Wei-Te Lei, Jiann-Jy Chen, Yow-Ling Shiue, Kuan-Pin Su, Chih-Sung Liang, Ping-Tao Tseng

**Affiliations:** a Division of General Surgery, Department of Surgery, E-Da Cancer Hospital, I-Shou University, Kaohsiung, Taiwan; b School of Medicine, College of Medicine, I-Shou University, Kaohsiung, Taiwan; c Institute of Biomedical Sciences, National Sun Yat-sen University, Kaohsiung, Taiwan; d Department of Internal Medicine, E-Da Dachang Hospital, I-Shou University, Kaohsiung, Taiwan; e Department of Psychiatry, Kaohsiung Chang Gung Memorial Hospital and Chang Gung University College of Medicine, Kaohsiung, Taiwan; f Division of Hematology and Oncology, Department of Internal Medicine, Tri-Service General Hospital; School of Medicine, National Defense Medical University, Taipei, Taiwan; g School of Medicine, National Defense Medical Center, Taipei, Taiwan; h Department of Emergency Medicine, E-Da Dachang Hospital, I-Shou University, Kaohsiung, Taiwan; i School of Medicine for International Students, I-Shou University, Kaohsiung, Taiwan; j Innovation in Mental and Physical Health and Clinical Treatment (IMPACT) Strategic Research Centre, School of Medicine, Barwon Health, Deakin University, Geelong, VIC, Australia; k Department of Psychological Medicine, Institute of Psychiatry, Psychology and Neuroscience, King’s College London, London, UK; l Department of Sport, University of Vienna, Vienna, Austria; m Prospect Clinic for Otorhinolaryngology & Neurology, Kaohsiung, Taiwan; n Department of Psychiatry, Tri-Service General Hospital, Taipei, Taiwan; o Department of Psychiatry, College of Medicine, National Defense Medical University, Taipei, Taiwan; p Section of Immunology, Rheumatology, and Allergy Department of Pediatrics, Hsinchu Munipical MacKay Children’s Hospital, Hsinchu, Taiwan; q Center for Molecular and Clinical Immunology, Chang Gung University, Taoyuan, Taiwan; r Department of Otorhinolaryngology, E-Da Cancer Hospital, I-Shou University, Kaohsiung, Taiwan; s Institute of Precision Medicine, National Sun Yat-sen University, Kaohsiung, Taiwan; t Mind-Body Interface Research Center (MBI-Lab), China Medical University Hospital, Taichung, Taiwan; u College of Medicine, China Medical University, Taichung, Taiwan; v An-Nan Hospital, China Medical University, Tainan, Taiwan; w Department of Psychiatry, Beitou Branch, Tri-Service General Hospital; School of Medicine, National Defense Medical Center, Taipei, Taiwan; x Department of Psychiatry, National Defense Medical Center, Taipei, Taiwan; y Department of Psychology, College of Medical and Health Science, Asia University, Taichung, Taiwan

**Keywords:** colorectal malignancy, colorectal tumor, GLP-1 receptor agonist, network meta-analysis, semaglutide, SGLT2 inhibitor

## Abstract

**Background:**

Recent evidence has raised concerns about potential pro-oncogenic effects associated with glucagon-like peptide-1 (GLP-1) receptor agonists and sodium–glucose cotransporter 2 (SGLT2) inhibitors, particularly regarding gastrointestinal malignancies. Colorectal tumors, the third most diagnosed cancer globally, already show increased incidence in patients with metabolic disorders who typically require these medications. For example, obese subjects had a significantly higher risk of colorectal tumors than healthy subjects. However, existing evidence on this association remains inconsistent. This network meta-analysis (NMA) evaluated the comparative incidence of colorectal tumors associated with specific GLP-1 receptor agonists and SGLT2 inhibitors.

**Materials and Methods:**

We conducted a confirmatory NMA focused specifically on colorectal tumor incidence as an adverse effect, following Cochrane methodological recommendations. We performed a frequentist-based NMA of randomized controlled trials (RCTs) evaluating GLP-1 receptor agonists or SGLT2 inhibitors. Our primary outcome was colorectal tumor incidence, with safety profiles assessed by dropout rates as a secondary outcome.

**Results:**

This NMA encompassing 68 RCTs with 207 200 participants found that only semaglutide was associated with increased incidence of colorectal tumors compared to controls. A dose-stratified analysis revealed that high-dose injectable semaglutide (2.4 mg/week) was the only regimen associated with increased incidence. Furthermore, when focusing on RCTs of obese subjects, the increased colorectal tumor rate related to semaglutide still existed. Neither other GLP-1 receptor agonists nor any SGLT2 inhibitors demonstrated significant associations with colorectal tumor development.

**Conclusion:**

Our study provides the first comprehensive NMA addressing the incidence of colorectal tumors related to individual GLP-1 receptor agonists and SGLT2 inhibitors, suggesting a dose–dependent relationship to semaglutide, particularly in its high-dose injectable form (2.4 mg/week). These findings represent a potential risk signal that requires further validation, given the already elevated baseline colorectal tumor risk in the target population, especially in subjects with obesity. Future research should focus on long-term follow-up studies to better characterize the mechanisms and clinical implications of this semaglutide-specific risk signal.

**What this adds to the existing literature:**

This network meta-analysis, based on 68 randomized controlled trials, suggested that only semaglutide, especially in highdose injectable form (2.4 mg/week), was associated with increased incidence of colorectal tumors compared to controls, especially in obese patients. Neither other GLP-1 receptor agonists nor any SGLT2 inhibitors demonstrated significant associations with colorectal tumor development.

**Learning points:**

Our study provides evidence regarding the increased incidence of colorectal tumors related to semaglutide in a dose dependent way (2.4 mg/week).

**HIGHLIGHTS**

- Recent evidence has raised concerns about potential pro-oncogenic effects associated with glucagon-like peptide-1 (GLP-1) receptor agonists and sodium–glucose cotransporter 2 (SGLT2) inhibitors, particularly regarding colorectal tumors, which were the third most commonly diagnosed cancer worldwide.
- This network meta-analysis of 68 randomized controlled trials (RCTs), including 207 200 participants, found that only semaglutide was associated with a significantly increased risk of colorectal tumors compared to controls.
- Dose-stratified analysis revealed that high-dose injectable semaglutide (2.4 mg/week) carried the highest risk.
- Furthermore, when focusing on RCTs of obese subjects, this increased colorectal tumor risk related to semaglutide still existed.
- Importantly, no other GLP-1 receptor agonists or SGLT2 inhibitors demonstrated significant associations with colorectal tumor development, suggesting this effect may be specific to semaglutide rather than a class effect.

## Introduction

Glucagon-like peptide-1 (GLP-1) receptor agonists and sodium–glucose cotransporter 2 (SGLT2) inhibitors have emerged as novel glucose-lowering agents, featuring mechanisms of action distinct from those of conventional treatments^[1]^. However, recently accumulating evidence raised concerns about their potential association with various tumor risks^[2,3]^. This pro-carcinogenetic concerns resulted from their anti-apoptotic effects via activation of protein kinase C-epsilon (PKCε) and extracellular signal-regulated kinase 1/2 (ERK1/2) pathways^[4]^, which played important roles in cell proliferation and anti-apoptosis^[5,6]^. GLP-1 receptor signaling has been implicated in regulating mucosal growth in the small intestine and colon, indicating its potential involvement in intestinal tumor development^[7]^. Echoing these concerns, several traditional pairwise meta-analyses were conducted to address the association between various tumors and prescription of such medications, such as breast tumor^[8]^, thyroid tumor^[9]^, and prostate tumor^[10]^.

On the other hand, seldom reports directly addressed the relationship between gastrointestinal tract tumor risks and GLP-1 receptor agonists and SGLT2 inhibitors use, which tumors accounted for 26% of the global cancer incidence and 35% of all cancer-related deaths^[11]^. Wang *et al*, by retrospective database search without randomized procedure to minimize the potential difference across experimental and control groups, noticed that the overall GLP-1 receptor agonists were associated with a lower risk of colorectal tumors, especially in obese participants^[12,13]^. Zhu *et al*, through immunological analyses of GLP-1 signaling-related genes in colorectal cancer, suggested that semaglutide might have protective roles in colorectal tumors^[14]^. Lin *et al* had addressed a phenomenon regarding the different cancer risk profiles related to individual GLP-1 receptor agonists in their review article^[15]^, which might reflect the hypothesis of “biased agonism and polymorphic variation” regarding the different activation of downstream signal transduction pathways in response to different extrinsic GLP-1 receptor agonists^[16]^. Furthermore, Flausino *et al* demonstrated a beneficial effect of SGLT2 inhibitors on the survival outcomes in gastrointestinal cancer patients undergoing chemotherapy/radiotherapy in their retrospective cohort study^[17]^. When a clinical trial is unable to reach a conclusion due to limitations in experimental design, meta-analyses can provide an alternative and important insight. By deriving data from numerous large-scale clinical studies or databases, they would be more reflective of clinical experience. Many scholars believe that meta-analyses based on large databases or numerous clinical trials can serve as a benchmark for guiding future research directions^[18,19]^. In 2024, Figlioli *et al* published a traditional pairwise meta-analysis, which suggested that GLP-1 receptor agonists did not alter overall gastrointestinal tumor risk but did not provide detailed information regarding site-specific tumors^[20]^. Similarly, Xu *et al* conducted another traditional pairwise meta-analysis addressing the association between SGLT2 inhibitors and several types of cancers and found that SGLT2 inhibitors did not alter gastrointestinal cancer risk^[21]^. However, as we know, the gastrointestinal tumor includes various kinds of tumors with different risk factors^[22]^ and epidemiologic data^[23]^. Among those gastrointestinal tumors, colorectal tumors were the third most diagnosed cancer in the world, with increasing rates in emerging economies^[24]^. The rationale of importance of exploring the association between colorectal tumors and GLP-1 receptor agonists and SGLT2 inhibitors prescription relied on two reasons. First, given the fact that subjects who needed such medications already had increased risks of colorectal tumors at baseline^[25]^, any medications with potential for pro-oncogenesis should be used with caution. Finally, the anti-apoptotic effects related to such medications were specifically noticed in colorectal tumors^[26,27]^. Therefore, the exploration of association between different risks of colorectal tumors and such medications was clinically relevant and important. However, to date, there have been few meta-analyses that addressed this issue. Specifically, Bushi and colleagues, by pooling different medications into one whole group, suggested that GLP-1 receptor agonists overall did not increase the risk of colorectal tumors^[28]^. However, given the fact that different medications would have different pharmacokinetic properties, the procedure of pooling different medications into one group would ignore the potential different tumor risk across individual medications.

To our knowledge, there was no network meta-analysis (NMA) in this field. Actually, a well-designed NMA, which allows for direct comparisons among different medications, could enhance the ability to make multiple treatment efficacy comparisons and assess the potential superiority of specific interventions at various dosages^[29]^. This approach offers a more detailed and evidence-based framework for guiding future clinical practices. Our current study follows the rationale of previously published studies on GLP-1 receptor agonists and SGLT2 inhibitors in the incidence of neurodegenerative disease^[30]^ and hearing loss^[31]^. Specifically, we conducted the current NMA to evaluate the potentially different risk of colorectal tumors related to GLP-1 receptor agonists and SGLT2 inhibitors prescription in subjects without baseline colorectal tumors.

## Methods

In this NMA, we used a confirmatory approach to specifically focus on particular adverse effects of interest (i.e., incidence of colorectal tumor here) based on the Cochrane recommendation^[32]^. This NMA adhered to the Preferred Reporting Items for Systematic Reviews and Meta-Analyses (PRISMA) guidelines^[33]^ (Supplementary Digital Content eTable 1, available at: http://links.lww.com/JS9/F134) and A MeaSurement Tool to Assess Systematic Reviews 2 guidelines^[34]^ (AMSTAR-2 checklist in Supplementary Digital Content Materials, available at: http://links.lww.com/JS9/F74). The current manuscript followed the TITAN Guidelines 2025 and the authors declared that there was no AI applied in the current work^[35]^ (Supplementary Digital Content eTable 9, available at: http://links.lww.com/JS9/F134).

### Database searches and study identification

We conducted comprehensive searches across multiple databases, including PubMed, Embase, ClinicalKey, Cochrane CENTRAL, ProQuest, ScienceDirect, Web of Science, and ClinicalTrials.gov (Supplementary Digital Content eTable 2, available at: http://links.lww.com/JS9/F73), covering publications up to 10 March 2025. Two independent researchers carried out these searches, screened titles and abstracts, and resolved disagreements through discussion. We also manually reviewed reference lists from relevant review articles and meta-analyses for additional studies^[20,28,36,37]^. No language restrictions were applied.

### Inclusion and exclusion criteria

This NMA was guided by the PICOS framework (Population, Intervention, Comparison, Outcome, Study Design): **Population:** Individuals without documented colorectal tumor at baseline; **Intervention:** Use of GLP-1 receptor agonists or SGLT2 inhibitors at any dose; **Comparison:** Placebo, standard care, or active comparator; **Outcome:** Incidence of colorectal tumor; **Study design**: Randomized controlled trials (RCTs).

RCTs exclusively enrolling participants with documented baseline colorectal tumors were excluded to maintain focus on causative or prophylactic evaluation. Furthermore, to minimize selective reporting bias, only RCTs with systematic adverse event assessments or targeted outcome evaluations were included^[38]^. Inclusion criteria were: (1) RCTs with participants free from documented colorectal tumor at baseline; (2) RCTs evaluating GLP-1 receptor agonists or SGLT2 inhibitors; (3) studies involving human subjects; and (4) RCTs with systematic adverse event monitoring or direct evaluation of target outcomes.

Exclusion criteria included: (1) non-RCT studies; (2) RCTs specifically enrolling participants with documented baseline colorectal tumors; (3) RCTs lacking direct comparisons of GLP-1 receptor agonists or SGLT2 inhibitors; (4) RCTs lacking information on target outcomes; and (5) animal studies.

### Methodological quality appraisal

Two independent reviewers assessed the risk of bias using the Cochrane Risk of Bias Tool 1.0^[39]^, achieving an inter-rater reliability score of 0.86. Discrepancies were resolved by consulting a third reviewer.

### Outcome definition

We recognized the fact that most RCTs were not specifically designed for detecting the incidence of colorectal tumors, so that they might not necessarily differentiate benign and malignant tumors clearly. Furthermore, the diagnostic criteria and cut-off points of benign tumors versus malignant cancers varied across countries and years. Finally, the colorectal tumor had a high percentage of transforming from a benign one into a malignant one depending on various clinical factors^[40,41]^. Therefore, we did not separate benign tumors and malignant cancers because of the limitation. Rather, we counted them together. Safety was evaluated through dropout rates, indicating study withdrawals for any reason.

### Data extraction, management, and conversion

Two independent authors extracted relevant data, including demographics, study design, treatment details, primary outcomes, and safety data. The corresponding authors were contacted for missing information. Data extraction followed protocols from the Cochrane Handbook for Systematic Reviews of Interventions and other medical literature standards^[42]^.

### Statistical analyses

A random-effects model was applied for analyses involving multiple treatment arms^[43]^, using MetaInsight (version 4.0.2, Complex Reviews Support Unit, National Institute for Health Research, London, UK) in a frequentist framework. MetaInsight uses the *netmeta* package in R for NMAs^[44]^.

For categorical data, single-zero-event studies received continuity correction, while studies with zero events in both treatment and control arms were excluded to avoid bias^[45,46]^. Forest plots were created to present odds ratios (ORs) with 95% confidence intervals (95% CIs) for effect size calculation^[47]^. We then generated treatment rankings and effect sizes for direct and indirect comparisons, tabulated accordingly. The “node-splitting” method was applied to test consistency between direct and indirect evidence, a method particularly beneficial in NMA when trial-level data are available^[44,48]^. A two-tailed *P*-value less than 0.05 indicated statistical significance.

### Heterogeneity, inconsistency, and sensitivity analyses

We evaluated heterogeneity among the included studies with the tau value, *I^2^*, and corresponding *P* value. The inconsistencies were explored by the loop-specific approach, node-splitting, and design-by-treatment model^[49]^. Furthermore, we used funnel plots and Egger’s regression to evaluate potentially publication bias. Because MetaInsight did not provide heterogeneity test, inconsistency test of loop-specific approach and design-by-treatment model, funnel plots, and Egger’s regression, we did these analyses with the *mvmeta* command in STATA (version 18.0)^[50]^. To assess the robustness of our findings, we conducted a sensitivity analysis of subgroup analysis by subgrouping treatments according to different dosages of GLP-1 receptor agonists or SGLT2 inhibitors. The dose definitions followed original RCT classifications^[51–116]^:

- **Canagliflozin:** Low: 100 mg; High: 300 mg.
- **Dapagliflozin**: Low: 2.5 mg; Medium: 5 mg; High: 10 mg.
- **Dulaglutide**: Low: <1.5 mg; Medium: 1.5 mg; High: >1.5 mg.
- **Efpeglenatide**: Low: 2 mg; Medium: 4 mg; High: 6 mg.
- **Empagliflozin**: Low: 1–10 mg; High: 25–50 mg.
- **Ertugliflozin**: Low: 5 mg; High: 15 mg.
- **Injectable semaglutide**: Low: 0.05–0.5 mg; Medium: 1.0 mg; High: 2.4 mg.
- **Tirzepatide**: Low 5 mg; Medium: 10 mg; High: 15 mg.

Finally, regarding the aforementioned different effects of GLP-1 receptor agonists on colorectal tumor risk in subjects with obesity^[12,13]^, we arranged a sensitivity analysis of subgroup analysis focusing on RCTs with obese subjects. Additionally, in consideration of the potential effect of medication exposure time on the incidence of colorectal tumor risks, we arranged a sensitivity analysis of subgroup analysis of RCTs with (1) less than 2-year treatment and (2) at least 2-year treatment. Finally, we evaluated the overall quality of evidence in our NMA with the GRADE^[117]^.

### General declaration

This study complies with the principles outlined in the Declaration of Helsinki.

## Results

### Eligibility of the studies

**Figure 1.**
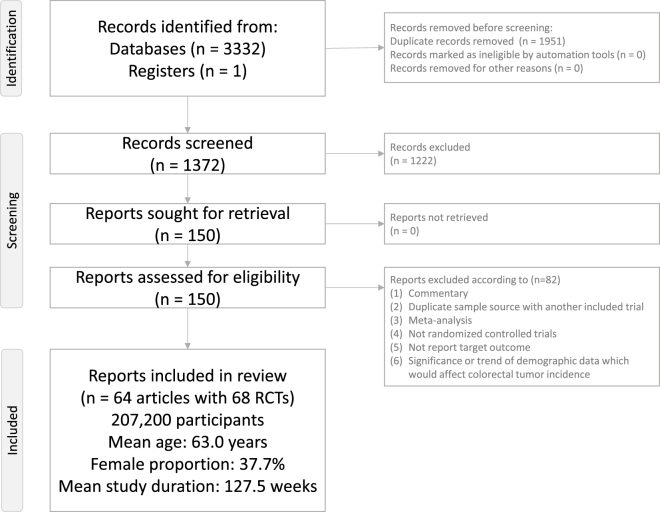
PRISMA2020 flowchart of current network meta-analysis.

Figure 1 illustrates the flowchart summarizing the literature search and screening process for this NMA. After excluding 82 articles for various reasons (Supplementary Digital Content eTable 3, available at: http://links.lww.com/JS9/F134), a total of 64 articles encompassing 68 RCTs were included in the analysis (Supplementary Digital Content eTable 4, available at: http://links.lww.com/JS9/F134)^[51–116]^. The selected studies involved 207200 participants (mean age = 63.0 years, range: 45.1–71.9 years; mean female proportion = 37.7%, range: 20.8–78.5%). The average study duration was 127.5 weeks (range: 24–281 weeks). The GLP-1 receptor agonists investigated included tirzepatide, efpeglenatide, liraglutide, albiglutide, dulaglutide, exenatide, semaglutide, and lixisenatide. The SGLT2 inhibitors investigated included bexagliflozin, canagliflozin, empagliflozin, ertugliflozin, dapagliflozin, and sotagliflozin.

### Primary outcome: overall colorectal tumor events

**Figure 2.**
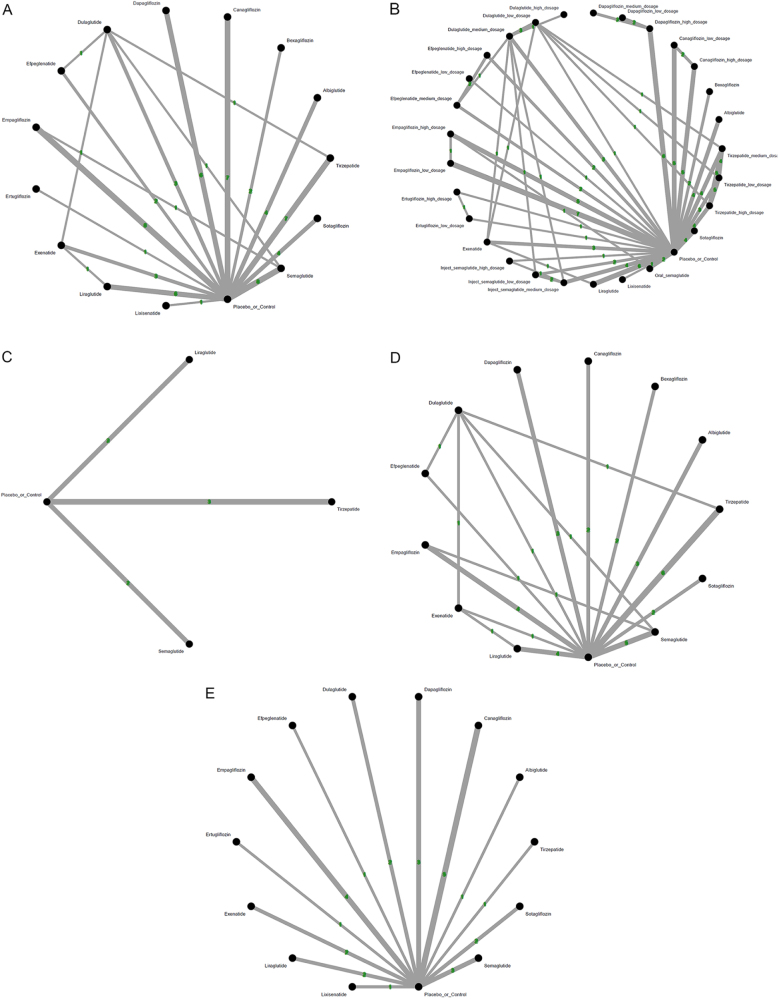
Network structure of the primary outcome: (A) overall colorectal tumor events, (B) colorectal tumor events in aspect of various dosage subgroups, (C) colorectal tumor events in RCTs of subjects with obesity, (D) colorectal tumor events in RCTs of treatment duration less than 2 years, and (E) colorectal tumor events in RCTs of treatment duration at least 2 years.

The main results of the primary outcome revealed that semaglutide was the only treatment associated with a statistically significant increased incidence of colorectal tumor [OR = 1.49, 95% CIs = 1.01–2.19, absolute risk difference (ARD) = 0.13%, number needed to harm (NNH) = 772] in comparison with the controls. On the other hand, bexagliflozin was associated with significantly less incidence of colorectal tumor than semaglutide (OR = 0.14, 95% CIs = 0.02–0.91) and sotagliflozin (OR = 0.09, 95% CIs = 0.01–0.73), respectively (Fig. 2A, Fig. 3A, Supplementary Digital Content eFigure 3A, available at: http://links.lww.com/JS9/F73, and Table 1).

The overall structure of the network meta-analysis. The lines between nodes represent direct comparisons from various trials, with the numbers over the lines indicating the number of trials providing these comparisons for each specific treatment. The thickness of the lines corresponds to the number of trials linked to the network.

**Figure 3.**
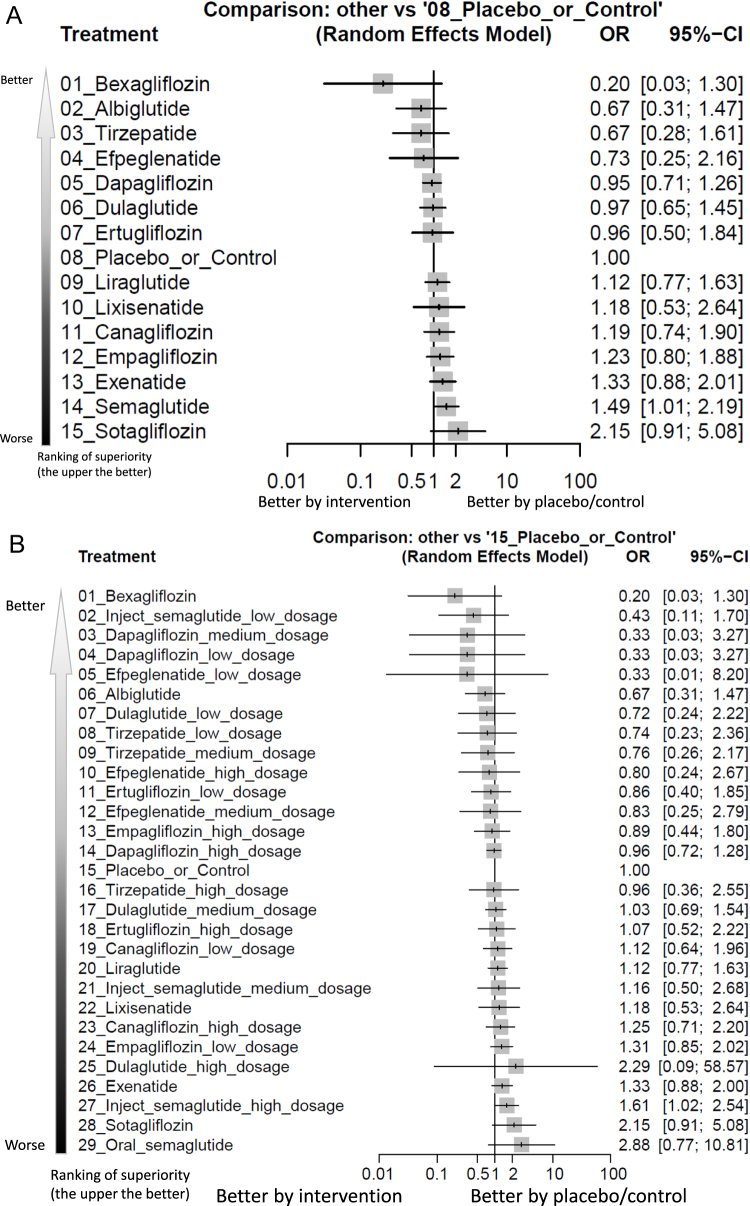
Forest plot of primary outcome: (A) overall colorectal tumor events, (B) colorectal tumor events in aspect of various dosage subgroups, (C) colorectal tumor events in RCTs of subjects with obesity, (D) colorectal tumor events in RCTs of treatment duration less than 2 years, and (E) colorectal tumor events in RCTs of treatment duration at least 2 years.

When the effect size (expressed as odds ratio) is less than 1, the specified treatment is associated with fewer colorectal tumor events compared to placebo/controls. 95% CIs, 95% confidence intervals; GLP-1 agonist, glucagon-like peptide-1 agonist; NMA, network meta-analysis; OR, odds ratio; RCT, randomized controlled trial; SGLT2 inhibitor, sodium–glucose cotransporter 2 inhibitor.

**Figure 3.**
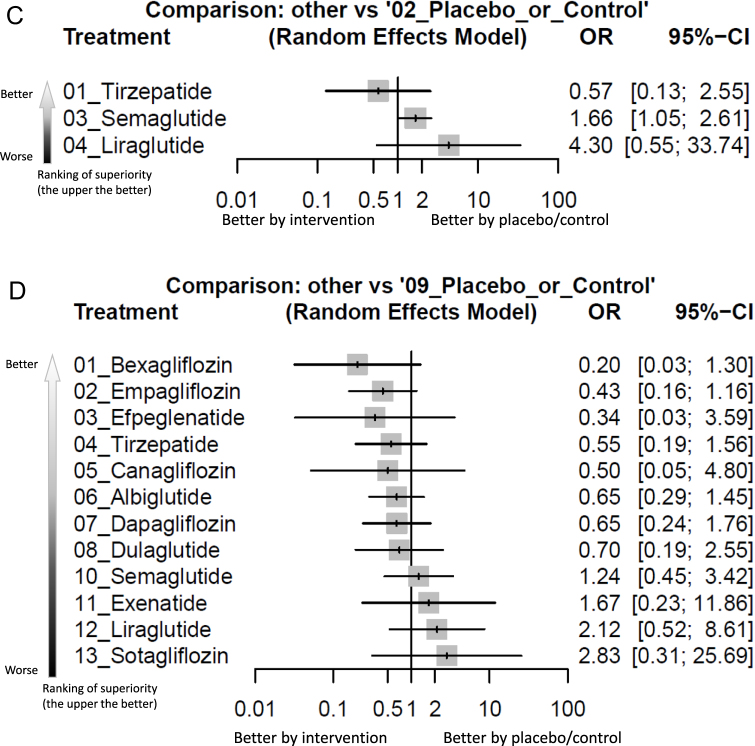

**Figure 3.**
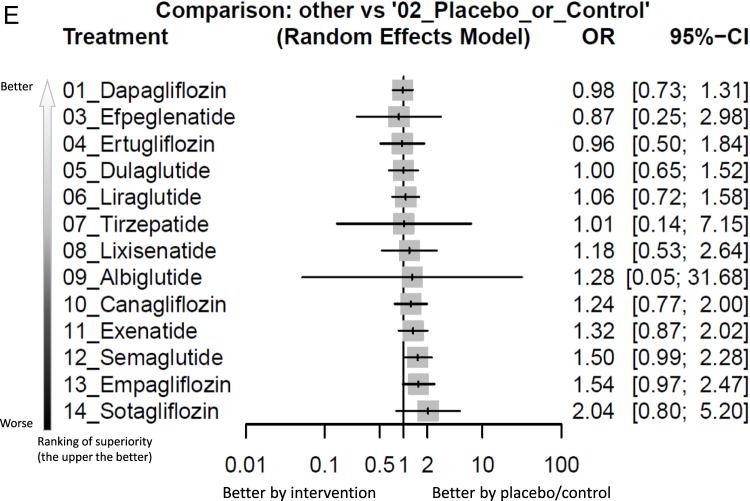

**Table 1:** League table of the primary outcome: overall colorectal tumor events

Bexagliflozin	.	.	.	.	.	.	0.20 [0.03; 1.30]	.	.	.	.	.	.	.
0.30 [0.04; 2.27]	Albiglutide	.	.	.	.	.	0.67 [0.31; 1.47]	.	.	.	.	.	.	.
0.30 [0.04; 2.37]	1.01 [0.31; 3.26]	Tirzepatide	.	.	0.50 [0.08; 3.00]	.	0.74 [0.27; 2.00]	.	.	.	.	.	.	.
0.28 [0.03; 2.39]	0.92 [0.24; 3.50]	0.91 [0.23; 3.67]	Efpeglenatide	.	1.50 [0.06; 36.96]	.	0.67 [0.21; 2.11]	.	.	.	.	.	.	.
0.21 [0.03; 1.41]	0.71 [0.31; 1.64]	0.71 [0.28; 1.78]	0.77 [0.25; 2.37]	Dapagliflozin	.	.	0.95 [0.71; 1.26]	.	.	.	.	.	.	.
0.21 [0.03; 1.40]	0.69 [0.29; 1.67]	0.69 [0.27; 1.73]	0.75 [0.24; 2.34]	0.97 [0.59; 1.59]	Dulaglutide	.	0.96 [0.63; 1.45]	.	.	.	.	0.17 [0.01; 4.07]	5.04 [0.24; 105.24]	.
0.21 [0.03; 1.51]	0.70 [0.25; 1.93]	0.69 [0.23; 2.06]	0.76 [0.21; 2.68]	0.98 [0.48; 1.99]	1.01 [0.47; 2.16]	Ertugliflozin	0.96 [0.50; 1.84]	.	.	.	.	.	.	.
0.20 [0.03; 1.30]	0.67 [0.31; 1.47]	0.67 [0.28; 1.61]	0.73 [0.25; 2.16]	0.95 [0.71; 1.26]	0.97 [0.65; 1.45]	0.96 [0.50; 1.84]	Placebo_or_Control	0.91 [0.62; 1.33]	0.85 [0.38; 1.89]	0.84 [0.52; 1.34]	0.80 [0.52; 1.23]	0.75 [0.50; 1.15]	*0.66 [0.44; 0.97]	0.47 [0.20; 1.10]
0.18 [0.03; 1.21]	0.60 [0.25; 1.44]	0.60 [0.23; 1.55]	0.65 [0.21; 2.06]	0.85 [0.53; 1.36]	0.87 [0.50; 1.51]	0.86 [0.41; 1.82]	0.89 [0.61; 1.31]	Liraglutide	.	.	.	2.97 [0.12; 73.39]	.	.
0.17 [0.02; 1.30]	0.57 [0.19; 1.75]	0.57 [0.17; 1.86]	0.62 [0.16; 2.38]	0.80 [0.34; 1.88]	0.82 [0.33; 2.02]	0.81 [0.29; 2.29]	0.85 [0.38; 1.89]	0.95 [0.39; 2.30]	Lixisenatide	.	.	.	.	.
0.17 [0.02; 1.16]	0.57 [0.23; 1.41]	0.56 [0.21; 1.52]	0.61 [0.19; 2.00]	0.79 [0.46; 1.37]	0.82 [0.44; 1.52]	0.81 [0.36; 1.80]	0.84 [0.52; 1.34]	0.94 [0.51; 1.72]	0.99 [0.39; 2.52]	Canagliflozin	.	.	.	.
0.17 [0.02; 1.11]	0.55 [0.22; 1.34]	0.54 [0.21; 1.44]	0.60 [0.19; 1.90]	0.77 [0.46; 1.28]	0.79 [0.44; 1.42]	0.78 [0.36; 1.70]	0.81 [0.53; 1.24]	0.91 [0.52; 1.61]	0.96 [0.39; 2.39]	0.97 [0.52; 1.83]	Empagliflozin	.	0.33 [0.01; 8.21]	.
0.15 [0.02; 1.02]	0.51 [0.21; 1.23]	0.50 [0.19; 1.32]	0.55 [0.17; 1.75]	0.71 [0.43; 1.17]	0.73 [0.41; 1.30]	0.72 [0.34; 1.56]	0.75 [0.50; 1.14]	0.84 [0.48; 1.47]	0.89 [0.36; 2.20]	0.90 [0.48; 1.67]	0.92 [0.51; 1.67]	Exenatide	.	.
*0.14 [0.02; 0.91]	0.45 [0.19; 1.08]	0.45 [0.17; 1.17]	0.49 [0.16; 1.55]	0.63 [0.39; 1.02]	0.65 [0.38; 1.13]	0.65 [0.30; 1.37]	*0.67 [0.46; 0.99]	0.75 [0.44; 1.29]	0.79 [0.33; 1.94]	0.80 [0.43; 1.47]	0.82 [0.47; 1.45]	0.89 [0.51; 1.57]	Semaglutide	.
*0.09 [0.01; 0.73]	0.31 [0.10; 1.00]	0.31 [0.09; 1.06]	0.34 [0.09; 1.35]	0.44 [0.18; 1.09]	0.45 [0.18; 1.17]	0.45 [0.15; 1.32]	0.47 [0.20; 1.10]	0.52 [0.20; 1.33]	0.55 [0.17; 1.79]	0.55 [0.21; 1.48]	0.57 [0.22; 1.49]	0.62 [0.24; 1.61]	0.69 [0.27; 1.78]	Sotagliflozin

95% CIs, 95% confidence intervals; GLP-1 agonist, glucagon-like peptide-1 agonist; NMA, network meta-analysis; OR, odds ratio; RCT, randomized controlled trial; SGLT2 inhibitor, sodium–glucose cotransporter 2 inhibitor.

Data present as OR [95% CIs]. Pairwise (upper-right portion) and network (lower-left portion) meta-analysis results are presented as estimated effect sizes for the outcome of overall events of colorectal tumors. Interventions are reported in order of mean ranking of beneficially prophylactic effect on overall events of colorectal tumors, and outcomes are expressed as OR (95% CIs). For the pairwise meta-analyses, OR of less than 1 indicates that the treatment specified in the row obtained more beneficial effect than that specified in the column. For the NMA, OR of less than 1 indicates that the treatment specified in the column obtained more beneficial effect than that specified in the row. Bold results marked with * indicate statistical significance.

*Sensitivity analysis of subgroup analysis of colorectal tumor events in aspect of various dosage subgroups*

In our analysis of subgroups according to various dosages, high-dose injectable semaglutide (2.4 mg/week) was the only regimen associated with a significantly increased incidence of colorectal tumors (OR = 1.61, 95% CIs = 1.02–2.54, ARD = 0.23%, NNH = 426) compared to control. On the other hand, bexagliflozin was associated with significantly less incidence of colorectal tumor than high-dose injectable semaglutide (2.4 mg/week) (OR = 0.13, 95% CIs = 0.02–0.85), sotagliflozin (OR = 0.09, 95% CIs = 0.01–0.73), and oral semaglutide (OR = 0.07, 95% CIs = 0.01–0.69), respectively (Fig. 2B, Fig. 3B, Supplementary Digital Content eFigure 3B, available at: http://links.lww.com/JS9/F134, and Table 2).

**Table 2:** League table of the primary outcome: colorectal tumor events in aspect of various dosage subgroups

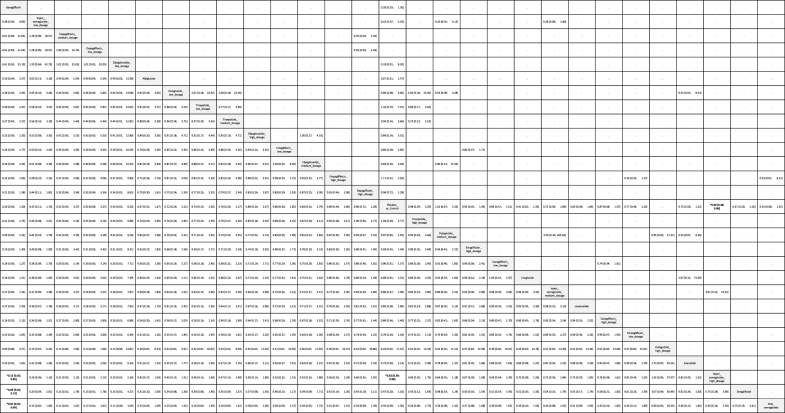

95% CIs, 95% confidence intervals; GLP-1 agonist, glucagon-like peptide-1 agonist; NMA, network meta-analysis; OR, odds ratio; RCT, randomized controlled trial; SGLT2 inhibitor, sodium–glucose cotransporter 2 inhibitor.

Data present as OR [95% CIs]. Pairwise (upper-right portion) and network (lower-left portion) meta-analysis results are presented as estimated effect sizes for the outcome of events of colorectal tumors in aspect of various dosage subgroups. Interventions are reported in order of mean ranking of beneficially prophylactic effect on events of colorectal tumors in aspect of various dosage subgroups, and outcomes are expressed as OR (95% CIs). For the pairwise meta-analyses, OR of less than 1 indicates that the treatment specified in the row obtained more beneficial effect than that specified in the column. For the NMA, OR of less than 1 indicates that the treatment specified in the column obtained more beneficial effect than that specified in the row. Bold results marked with * indicate statistical significance.

*Sensitivity analysis of subgroup analysis of colorectal tumor events in RCTs of obese subjects*

In our analysis of subgroups focusing on RCTs on obese subjects, only semaglutide was associated with a significantly increased incidence of colorectal tumors (OR = 1.66, 95% CIs = 1.05–2.61, ARD = 0.19%, NNH = 513) compared to control (Fig. 2C, Fig. 3C, and Table 3).

**Table 3:** League table of the primary outcome: colorectal tumor events in RCTs of subjects with obesity

Tirzepatide	0.57 [0.13; 2.55]	.	.
0.57 [0.13; 2.55]	Placebo_or_Control	*0.60 [0.38; 0.95]	0.23 [0.03; 1.83]
0.34 [0.07; 1.65]	*0.60 [0.38; 0.95]	Semaglutide	.
0.13 [0.01; 1.69]	0.23 [0.03; 1.83]	0.39 [0.05; 3.18]	Liraglutide

95% CIs, 95% confidence intervals; GLP-1 agonist, glucagon-like peptide-1 agonist; NMA, network meta-analysis; OR, odds ratio; RCT, randomized controlled trial; SGLT2 inhibitor, sodium–glucose cotransporter 2 inhibitor.

Data present as OR [95% CIs]. Pairwise (upper-right portion) and network (lower-left portion) meta-analysis results are presented as estimated effect sizes for the outcome of events of colorectal tumors in RCTs of subjects with obesity. Interventions are reported in order of mean ranking of beneficially prophylactic effect on events of colorectal tumors in RCTs of subjects with obesity, and outcomes are expressed as OR (95% CIs). For the pairwise meta-analyses, OR of less than 1 indicates that the treatment specified in the row obtained more beneficial effect than that specified in the column. For the NMA, OR of less than 1 indicates that the treatment specified in the column obtained more beneficial effect than that specified in the row. Bold results marked with * indicate statistical significance.

*Sensitivity analysis of subgroup analysis of colorectal tumor events in RCTs of “less than 2-year treatment” and “at least 2-year treatment”*

In the subgroup analysis of “less than 2-year treatment,” none of the investigated medications was associated with a significantly different risk of colorectal tumor in comparison with controls (Fig. 2D, Fig. 3D, and Table 4). Rather, in subgroup analysis of “at least 2-year treatment,” only semaglutide achieved a trend of increased risk of colorectal tumor (OR = 1.50, 95% CIs = 0.99–2.28) compared to control (Fig. 2E, Fig. 3E, and Table 5).

**Table 4:** League table of the primary outcome: colorectal tumor events in RCTs of treatment duration less than 2 years

Bexagliflozin	.	.	.	.	.	.	.	0.20 [0.03; 1.30]	.	.	.	.
0.47 [0.06; 3.90]	Empagliflozin	.	.	.	.	.	.	0.43 [0.15; 1.23]	0.33 [0.01; 8.21]	.	.	.
0.60 [0.03; 12.02]	1.26 [0.10; 16.28]	Efpeglenatide	.	.	.	.	1.50 [0.06; 36.96]	0.11 [0.00; 2.71]	.	.	.	.
0.37 [0.04; 3.12]	0.78 [0.18; 3.30]	0.62 [0.05; 7.41]	Tirzepatide	.	.	.	0.50 [0.08; 3.00]	0.66 [0.21; 2.11]	.	.	.	.
0.41 [0.02; 7.63]	0.86 [0.07; 10.25]	0.68 [0.03; 17.96]	1.10 [0.09; 13.36]	Canagliflozin	.	.	.	0.50 [0.05; 4.80]	.	.	.	.
0.31 [0.04; 2.38]	0.66 [0.18; 2.40]	0.53 [0.04; 6.36]	0.85 [0.23; 3.18]	0.77 [0.07; 8.55]	Albiglutide	.	.	0.65 [0.29; 1.45]	.	.	.	.
0.31 [0.04; 2.59]	0.66 [0.16; 2.73]	0.53 [0.04; 6.81]	0.85 [0.20; 3.61]	0.77 [0.06; 9.18]	1.00 [0.28; 3.63]	Dapagliflozin	.	0.65 [0.24; 1.76]	.	.	.	.
0.29 [0.03; 2.79]	0.61 [0.12; 3.10]	0.49 [0.05; 5.13]	0.78 [0.21; 2.98]	0.71 [0.05; 9.67]	0.92 [0.20; 4.24]	0.92 [0.18; 4.74]	Dulaglutide	0.25 [0.02; 2.77]	5.04 [0.24; 105.24]	0.17 [0.01; 4.07]	.	.
0.20 [0.03; 1.30]	0.43 [0.16; 1.16]	0.34 [0.03; 3.59]	0.55 [0.19; 1.56]	0.50 [0.05; 4.80]	0.65 [0.29; 1.45]	0.65 [0.24; 1.76]	0.70 [0.19; 2.55]	Placebo_or_Control	0.60 [0.20; 1.85]	0.65 [0.03; 16.15]	0.57 [0.13; 2.54]	0.35 [0.04; 3.20]
0.16 [0.02; 1.36]	0.35 [0.09; 1.34]	0.27 [0.02; 3.45]	0.44 [0.11; 1.83]	0.40 [0.03; 4.82]	0.52 [0.14; 1.91]	0.52 [0.13; 2.18]	0.56 [0.12; 2.60]	0.81 [0.29; 2.23]	Semaglutide	.	.	.
0.12 [0.01; 1.82]	0.26 [0.03; 2.32]	0.20 [0.01; 3.98]	0.33 [0.04; 2.81]	0.30 [0.01; 5.99]	0.39 [0.05; 3.24]	0.39 [0.04; 3.52]	0.42 [0.05; 3.37]	0.60 [0.08; 4.27]	0.74 [0.08; 6.58]	Exenatide	0.34 [0.01; 8.30]	.
*0.10 [0.01; 0.98]	0.20 [0.04; 1.13]	0.16 [0.01; 2.44]	0.26 [0.05; 1.46]	0.23 [0.02; 3.37]	0.30 [0.06; 1.54]	0.30 [0.05; 1.71]	0.33 [0.05; 2.10]	0.47 [0.12; 1.92]	0.58 [0.10; 3.27]	0.79 [0.10; 6.46]	Liraglutide	.
0.07 [0.00; 1.28]	0.15 [0.01; 1.70]	0.12 [0.00; 3.03]	0.19 [0.02; 2.22]	0.18 [0.01; 4.15]	0.23 [0.02; 2.39]	0.23 [0.02; 2.57]	0.25 [0.02; 3.18]	0.35 [0.04; 3.20]	0.44 [0.04; 4.95]	0.59 [0.03; 11.25]	0.75 [0.05; 10.20]	Sotagliflozin

95% CIs, 95% confidence intervals; GLP-1 agonist, glucagon-like peptide-1 agonist; NMA, network meta-analysis; OR, odds ratio; RCT, randomized controlled trial; SGLT2 inhibitor, sodium–glucose cotransporter 2 inhibitor.

Data present as OR [95% CIs]. Pairwise (upper-right portion) and network (lower-left portion) meta-analysis results are presented as estimated effect sizes for the outcome of events of colorectal tumors in RCTs of subjects with obesity. Interventions are reported in order of mean ranking of beneficially prophylactic effect on events of colorectal tumors in RCTs of subjects with obesity, and outcomes are expressed as OR (95% CIs). For the pairwise meta-analyses, OR of less than 1 indicates that the treatment specified in the row obtained more beneficial effect than that specified in the column. For the NMA, OR of less than 1 indicates that the treatment specified in the column obtained more beneficial effect than that specified in the row. Bold results marked with * indicate statistical significance.

**Table 5:** League table of the primary outcome: colorectal tumor events in RCTs of treatment duration at least 2 years

Dapagliflozin	0.98 [0.73; 1.31]	.	.	.	.	.	.	.	.	.	.	.	.
0.98 [0.73; 1.31]	Placebo_or_Control	1.15 [0.34; 3.92]	1.04 [0.54; 1.98]	1.00 [0.66; 1.54]	0.94 [0.63; 1.40]	0.99 [0.14; 7.08]	0.85 [0.38; 1.89]	0.78 [0.03; 19.31]	0.81 [0.50; 1.31]	0.76 [0.50; 1.16]	0.66 [0.44; 1.01]	0.65 [0.41; 1.03]	0.49 [0.19; 1.24]
1.12 [0.32; 3.97]	1.15 [0.34; 3.92]	Efpeglenatide	.	.	.	.	.	.	.	.	.	.	.
1.01 [0.50; 2.07]	1.04 [0.54; 1.98]	0.91 [0.23; 3.63]	Ertugliflozin	.	.	.	.	.	.	.	.	.	.
0.98 [0.58; 1.65]	1.00 [0.66; 1.54]	0.88 [0.24; 3.22]	0.97 [0.45; 2.10]	Dulaglutide	.	.	.	.	.	.	.	.	.
0.92 [0.56; 1.50]	0.94 [0.63; 1.40]	0.82 [0.23; 2.98]	0.91 [0.42; 1.93]	0.94 [0.52; 1.67]	Liraglutide	.	.	.	.	.	.	.	.
0.97 [0.13; 7.07]	0.99 [0.14; 7.08]	0.87 [0.09; 8.79]	0.96 [0.12; 7.57]	0.99 [0.13; 7.38]	1.06 [0.14; 7.83]	Tirzepatide	.	.	.	.	.	.	.
0.83 [0.35; 1.95]	0.85 [0.38; 1.89]	0.74 [0.17; 3.21]	0.81 [0.29; 2.29]	0.84 [0.34; 2.09]	0.90 [0.37; 2.20]	0.85 [0.10; 7.08]	Lixisenatide	.	.	.	.	.	.
0.76 [0.03; 19.13]	0.78 [0.03; 19.31]	0.68 [0.02; 21.14]	0.75 [0.03; 19.84]	0.78 [0.03; 19.78]	0.83 [0.03; 21.04]	0.78 [0.02; 33.71]	0.92 [0.03; 25.22]	Albiglutide	.	.	.	.	.
0.79 [0.45; 1.39]	0.81 [0.50; 1.31]	0.70 [0.19; 2.64]	0.78 [0.35; 1.74]	0.80 [0.42; 1.53]	0.86 [0.46; 1.60]	0.81 [0.11; 6.12]	0.96 [0.37; 2.44]	1.03 [0.04; 26.51]	Canagliflozin	.	.	.	.
0.74 [0.44; 1.24]	0.76 [0.50; 1.16]	0.66 [0.18; 2.42]	0.73 [0.34; 1.58]	0.75 [0.41; 1.37]	0.80 [0.45; 1.44]	0.76 [0.10; 5.66]	0.90 [0.36; 2.22]	0.97 [0.04; 24.64]	0.94 [0.49; 1.78]	Exenatide	.	.	.
0.65 [0.39; 1.08]	0.66 [0.44; 1.01]	0.58 [0.16; 2.13]	0.64 [0.30; 1.38]	0.66 [0.37; 1.20]	0.71 [0.40; 1.26]	0.67 [0.09; 4.97]	0.79 [0.32; 1.95]	0.85 [0.03; 21.64]	0.82 [0.44; 1.56]	0.88 [0.49; 1.59]	Semaglutide	.	.
0.63 [0.36; 1.10]	0.65 [0.41; 1.03]	0.56 [0.15; 2.11]	0.62 [0.28; 1.39]	0.64 [0.34; 1.21]	0.69 [0.37; 1.27]	0.65 [0.09; 4.89]	0.77 [0.30; 1.94]	0.83 [0.03; 21.22]	0.80 [0.41; 1.57]	0.86 [0.46; 1.61]	0.97 [0.52; 1.82]	Empagliflozin	.
0.48 [0.18; 1.27]	0.49 [0.19; 1.24]	0.43 [0.09; 2.00]	0.47 [0.15; 1.47]	0.49 [0.17; 1.36]	0.52 [0.19; 1.43]	0.49 [0.06; 4.32]	0.58 [0.17; 1.98]	0.63 [0.02; 17.70]	0.61 [0.21; 1.73]	0.65 [0.23; 1.80]	0.74 [0.26; 2.04]	0.76 [0.27; 2.15]	Sotagliflozin

95% CIs, 95% confidence intervals; GLP-1 agonist, glucagon-like peptide-1 agonist; NMA, network meta-analysis; OR, odds ratio; RCT, randomized controlled trial; SGLT2 inhibitor, sodium–glucose cotransporter 2 inhibitor.

Data present as OR [95% CIs]. Pairwise (upper-right portion) and network (lower-left portion) meta-analysis results are presented as estimated effect sizes for the outcome of events of colorectal tumors in RCTs of subjects with obesity. Interventions are reported in order of mean ranking of beneficially prophylactic effect on events of colorectal tumors in RCTs of subjects with obesity, and outcomes are expressed as OR (95% CIs). For the pairwise meta-analyses, OR of less than 1 indicates that the treatment specified in the row obtained more beneficial effect than that specified in the column. For the NMA, OR of less than 1 indicates that the treatment specified in the column obtained more beneficial effect than that specified in the row. Bold results marked with * indicate statistical significance.

### Safety profile: dropout rate

The tirzepatide (OR = 0.61, 95% CIs = 0.50–0.74), canagliflozin (OR = 0.73, 95% CIs = 0.60–0.89), dulaglutide (OR = 0.77, 95% CIs = 0.63–0.95), liraglutide (OR = 0.80, 95% CIs = 0.68–0.95), semaglutide (OR = 0.81, 95% CIs = 0.68–0.97), and empagliflozin (OR = 0.85, 95% CIs = 0.74–0.97) were associated with significantly less dropout rates than the control group did. Among these interventions, tirzepatide ranked the best (Supplementary Digital Content eFigure 1, available at: http://links.lww.com/JS9/F134, Supplementary Digital Content eFigure 2, available at: http://links.lww.com/JS9/F134, Supplementary Digital Content eFigure 3C, available at: http://links.lww.com/JS9/F134, and Supplementary Digital Content eTable 5, available at: http://links.lww.com/JS9/F134).

### Publication bias, risk of bias, inconsistency, heterogeneity, and quality of evidence

Funnel plots of publication bias across the included studies revealed a general symmetry, and no significant publication bias was detected among the articles included in NMA by using the Egger test (Supplementary Digital Content eFigures 4A–4L, available at: http://links.lww.com/JS9/F134). We identified that 77.1% (367/476 items), 15.5% (74/476 items), and 7.4% (35/476 items) of the included studies had low, unclear, and high risks of bias, respectively (Supplementary Digital Content eFigures 5A–5B, available at: http://links.lww.com/JS9/F134). The inconsistency test, measured by loop-specific approach, node-splitting, and design-by-treatment model, showed no significant inconsistencies in the present NMA (Supplementary Digital Content eTable 6A–6G, available at: http://links.lww.com/JS9/F134). There was no significant heterogeneity detected within the NMA (Supplementary Digital Content eTable 7A–7F, available at: http://links.lww.com/JS9/F134). The overall quality of evidence of this NMA falls within moderate-high (Supplementary Digital Content eTable 8A–8C, available at: http://links.lww.com/JS9/F134).

## Discussion

To our knowledge, this is the first NMA to directly address the potential impact on colorectal tumor risk related to GLP-1 receptor agonists and SGLT2 inhibitors use in a dose–dependent way. In our NMA, we noticed that overall semaglutide within the recommended dosage was associated with a significantly increased incidence of colorectal tumors in a dose–dependent way, especially high-dose injectable semaglutide (2.4 mg/week) in subjects with obesity. Furthermore, even after subgrouping the included studies based on a treatment duration of at least 2 years, a trend toward an increased risk of colorectal tumors associated with semaglutide use remained, despite the reduced number of studies. Conversely, other investigated regimens were not associated with significantly different risks of colorectal tumors compared with controls.

The most important finding was that semaglutide was associated with a significantly increased risk of colorectal tumors in a dose–dependent way, which was different from the findings of previous traditional pairwise meta-analyses. In an earlier meta-analysis by Figlioli and colleagues, the authors pooled different kinds of gastrointestinal tract tumors into a whole group, ignoring the different site-specific tumor risk factors, and achieved results that overall GLP-1 receptor agonists would not alter the risk of overall gastrointestinal tract tumors^[20]^. Following that, although another traditional pairwise meta-analysis specifically focused on colorectal tumors, the authors were still limited to a methodology restriction, which was to pool all medications with different mechanisms into one group, and achieved results that overall GLP-1 receptor agonists did not significantly increase the colorectal tumor risk without specific information regarding individual medications^[28]^. Finally, Nagendra *et al* specifically focused on one medication (i.e., semaglutide) and achieved the result that semaglutide did not increase the risk of pancreatic tumor or thyroid tumor. However, they did not evaluate the changes in colorectal tumor risk^[37]^. Different from those previous traditional pairwise meta-analyses, the current NMA, by exhaustively dividing different medications with various dosages into different groups, found the significantly increased risk of colorectal tumor by a specific regimen under a specific dosage. The NMA achieved its results based on direct evidence (i.e., semaglutide-control direct comparison) and indirect evidence [i.e., semaglutide–dulaglutide (another GLP-1 receptor agonist)–control and semaglutide–empagliflozin (one SGLT2 inhibitor)–control indirect comparison] in Figure 2A. Therefore, the significant difference between semaglutide and controls existed not only in direct pairwise comparison (right-upper part in Table 1) but also in overall NMA comparison (left-lower part in Table 1). This finding would provide insight into the clinical practice that (1) not every GLP-1 receptor agonist would be safe regarding the risk of colorectal tumor and (2) semaglutide should be used with caution about colorectal tumor risk when the dosage reaches an upper limit (i.e., 2.4 mg/week) in inject form. In addition to the main finding, the NMA had extra merits providing multiple comparisons between various GLP-1 receptor agonists and SGLT2 inhibitors in the left-lower part of Table 1.

The potential pro-tumorigenic effect of semaglutide might be supported by some *in vivo* and *in vitro* studies. As addressed before, semaglutide exerted its anti-apoptotic effect via activation of PKCε and ERK1/2-MAPK pathway^[4]^. Consequently, activated PKCε could promote the survival of lung cancer cells by suppressing apoptosis^[5]^. The overexpressed PKCε has been demonstrated in numerous tumor-derived cell lines and histopathology tumor specimens^[118]^. The overexpression of PKCε was also one of the key features of metastatic colonic tumor cells and may be linked to ras-modulated signal transduction leading to neoplastic transformation in colonic epithelium^[26]^. On the other hand, the activation of ERK1/2 would generally promote cell proliferation, in which dysregulation was a hallmark of many cancers^[6]^. Specifically, the dysregulated ERK1/2 and downstream mitogen-activated protein kinase (MAPK) activity was frequently seen in colorectal tumors^[27]^. The molecular structural modification on amino acid chain positions 2 and 28, where alanine and lysine are replaced by 2-aminoisobutyric acid and arginine, improved semaglutide’s better combination with albumin, enabled longer presence in the blood circulation, and prevented chemical breakdown by dipeptidyl peptidase-4^[119]^. This molecular-level difference could extend its half-life and allow longer stimulation on the MAPK/ERK pathway in colorectal cells. On the other hand, the increased dosage of injection-form semaglutide would increase the potential pro-tumorigenic signals in the targeted cells through its constant high concentration related to the injection-form designation^[120]^. Finally, different from the uniform reaction to intrinsic GLP-1, the GLP-1 receptor would, under different extrinsic GLP-1 receptor agonist stimulation, activate different downstream signal transduction pathways (the so-called biased agonism and polymorphic variation)^[16]^. That is one of the main reasons why various GLP-1 receptor agonists would have different adverse effect profiles. However, as addressed before, the hypothesis remained theoretical and lacked direct evidence to support. Future direct human trials should be warranted to provide more information regarding the risk of colorectal tumors related to semaglutide prescription, especially in high-dose inject form.

This NMA offers several methodological strengths that enhance the reliability and clinical utility of our findings. First, the NMA designation could enable statistical comparisons between different GLP-1 receptor agonists and SGLT2 inhibitors, providing more comprehensive evidence than traditional pairwise meta-analyses. Our rigorous methodology included exclusively focusing on RCTs, ensuring high-quality evidence while minimizing potential bias. By specifically excluding participants with pre-existing colorectal tumors, we were able to isolate true causative or prophylactic effects. Finally, our detailed subgroup analysis across various dosages and focusing on RCTs of subjects with obesity offer clinicians granular evidence to inform a dose–dependent risk related to a specific regimen.

Some limitations warrant consideration. First, the primary limitation relates to study duration; although the included trials averaged 127.5 weeks (range: 24–281 weeks) for study duration, this timeframe may be insufficient to fully capture the development of colorectal tumors. Although we arranged subgroup analysis based on study duration and noticed a trend of increased colorectal tumor risk in the subgroup of “at least 2-year treatment,” the concerns of insufficient medication exposure still existed in a relatively short follow-up duration. Second, our stringent focus on RCTs, while ensuring methodological rigor, potentially excluded valuable observational data from long-term cohort studies. Third, the most included RCTs were not specifically designed for detecting the incidence of colorectal tumors, so that the variation in diagnostic approaches across multi-country trials would present a notable limitation. The designation of original RCTs may have introduced heterogeneity in case identification of colorectal tumors, so that it might lead to challenges in establishing causality from secondary safety endpoints. Although meta-analyses may raise concerns about “inconsistencies in diagnostic tools,” the nature of these studies is rooted in large databases or numerous clinical studies, making them more reflective of clinical experience. Therefore, many meta-analyses on drug side effects^[121,122]^ have become a benchmark for guiding future research^[18,19]^. Finally, all the recruited RCTs were designed based on various underlying diseases (i.e., diabetes or obesity) but not specifically to examine colorectal tumor occurrence, so that they might not necessarily distinguish between benign and malignant tumors clearly. Rather, most of them provided data on “colorectal tumors” but did not specifically address “benign or malignant tumors.” Therefore, although we recognized the importance of distinguishing benign and malignant tumors, we could not arrange a specific analysis focusing on malignant colorectal tumors. These limitations suggest the need for longer-term studies specifically designed to assess the risk of colorectal tumors related to semaglutide use.

## Conclusion

This NMA, the first to assess colorectal tumor risk across GLP-1 agonists and SGLT2 inhibitors, suggested that semaglutide, particularly the high-dose injectable formulation (2.4 mg/week) in individuals with obesity – may be associated with a dose–dependent increase in colorectal tumor incidence. This is notable given patients’ elevated baseline risk. Accordingly, the signal should be interpreted cautiously and confirmed in further studies.

## Data Availability

All the data of the current study were available upon reasonable request to the corresponding authors.

## Research registration unique identifying number (UIN)

PROSPERO CRD420251007952.
